# Clinical impact of the right ventricular impairment in patients following transcatheter aortic valve replacement

**DOI:** 10.1038/s41598-024-52242-w

**Published:** 2024-01-20

**Authors:** Satoshi Higuchi, Yasuhide Mochizuki, Tadashi Omoto, Hidenari Matsumoto, Tomoaki Masuda, Kazuto Maruta, Atsushi Aoki, Toshiro Shinke

**Affiliations:** 1 Division of Cardiology, Department of Medicine, Showa University School of Medicine, Tokyo, Japan; 2 Division of Cardiovascular Surgery, Department of Surgery, Showa University School of Medicine, Tokyo, Japan

**Keywords:** Interventional cardiology, Cardiology, Medical research, Risk factors

## Abstract

The right ventricular (RV) impairment can predict clinical adverse events in patients following transcatheter aortic valve replacement (TAVR) for severe aortic stenosis (AS). Limited reports have compared impact of the left ventricular (LV) and RV disorders. This retrospective study evaluated two-year major adverse cardiac and cerebrovascular events (MACCE) in patients following TAVR for severe AS. RV sphericity index was calculated as the ratio between RV mid-ventricular and longitudinal diameters during the end-diastolic phase. Of 239 patients, 2-year MACCE were observed in 34 (14%). LV ejection fraction was 58 ± 11%. Tricuspid annular plane systolic excursion (TAPSE) and RV sphericity index were 20 ± 3 mm and 0.36 (0.31–0.39). Although the univariate Cox regression analysis demonstrated that both LV and RV parameters predicted the outcomes, LV parameters no longer predicted them after adjustment. Lower TAPSE (adjusted hazard ratio per 1 mm, 0.84; 95% confidence interval, 0.75–0.93) and higher RV sphericity index (adjusted hazard ratio per 0.1, 1.94; 95% confidence interval, 1.17–3.22) were adverse clinical predictors. In conclusion, the RV structural and functional disorders predict two-year MACCE, whereas the LV parameters do not. Impact of LV impairment can be attenuated after development of RV disorders.

## Introduction

Transcatherter aortic valve replacement (TAVR) has been emerged as alternative therapy for patients with severe aortic stenosis (AS) at high or prohibitive surgical risk^1^ and can provide better prognosis in such population. However, even after the procedure, major adverse cardiac and cerebrovascular events (MACCE) such as heart failure hospitalization may occur and result in impaired activity daily living, higher cost, higher incidences of mortality and morbidities^2^. It would be necessary to identify patients at a risk of clinical adverse events after TAVR for suppression of cost and improvement in prognosis.

According to current guidelines, surgical or transcatheter aortic valve replacement (AVR) is determined based on the demonstration of severe AS, presence or absence of symptoms related to AS, and left ventricular ejection fraction (LVEF)^3^. The AVR decision algorithm does not include cardiac structural and functional findings other than LVEF. Of cardiac impairment, the right heart structure and function, which have been largely underestimated in the left heart diseases^4^, have attracted attention of clinicians and researchers for the last few years. Indeed, both structural and functional impairment of the right heart predicted worse clinical outcomes in patients who underwent TAVR according to some recent studies^5–8^. The conception of staging classification based on the extra-aortic valve cardiac damage supports the importance of the right heart findings^9^ and may suggest that the right ventricular (RV) dysfunction reflects the advanced left ventricular (LV) dysfunction^10^. It should be clarified which, or both of the LV and RV characteristics is important to predict adverse clinical events. Understanding of any interactions between the LV and RV (or the left and right heart system) disorders may contribute to better patient selection for TAVR.

The present study aimed to (1) identify predictors related to the RV structure and function, (2) demonstrate whether the RV dysfunction may predict adverse clinical outcomes more accurately than that of the LV, and (3) investigate which LV parameters are associated with the RV findings in patients who underwent TAVR for severe AS.

## Methods

### Study population

The current study was conducted based on a single-center, retrospective registry. Treatment option, surgical aortic valve replacement or TAVR, was determined by an interdisciplinary heart team at our hospital, considering the severity of AS, echocardiographic findings, symptoms, comorbidities including frailty, and life expectancy. TAVR was selected for patients at high surgical risk or those who were not considered to be suitable candidates for surgery because they had coexisting conditions^11^.

### Inclusion and exclusion criteria

Consecutive patients following TAVR at our hospital between 2015 and 2022 were included. Patients who underwent TAVR in surgical aortic valve (SAV) for aortic regurgitation (AR) were excluded from the present analysis.

### Data collection

Patient characteristics including age, sex, comorbidities, medical history, laboratory data, echocardiographic findings, oral medication at discharge, and 2-year cardiovascular events were collected. The laboratory and echocardiographic data were obtained from an electronic medical record at the nearest day within the last month prior to TAVR. Sapien XT, Sapien 3 (Edwards Lifesciences, Irvine, CA, USA), Evolut R, Evolut Pro, or Evolut Pro Plus (Medtronic, Minneapolis, MN, USA) was selected for the procedure. The transfemoral, transapical, or transsubclavian/transaxillary approach was applied.

### Study endpoint

This study assessed 2-year MACCE, a composite of cardiovascular death, hospitalization for heart failure, nonfatal acute myocardial infarction, and cerebral stroke. The follow-up was completed on the last medical interview date, last examination date, or date when an endpoint event was observed, whichever came first. Patients who become lost to follow-up at 2 years were censored. All data concerning the follow-up duration and adverse clinical events at 2 years were acquired from medical records or telephone interview. MACCE was confirmed by dedicated cardiologists that adjudicates all clinical events.

### Echocardiographic findings

**Figure 1:**
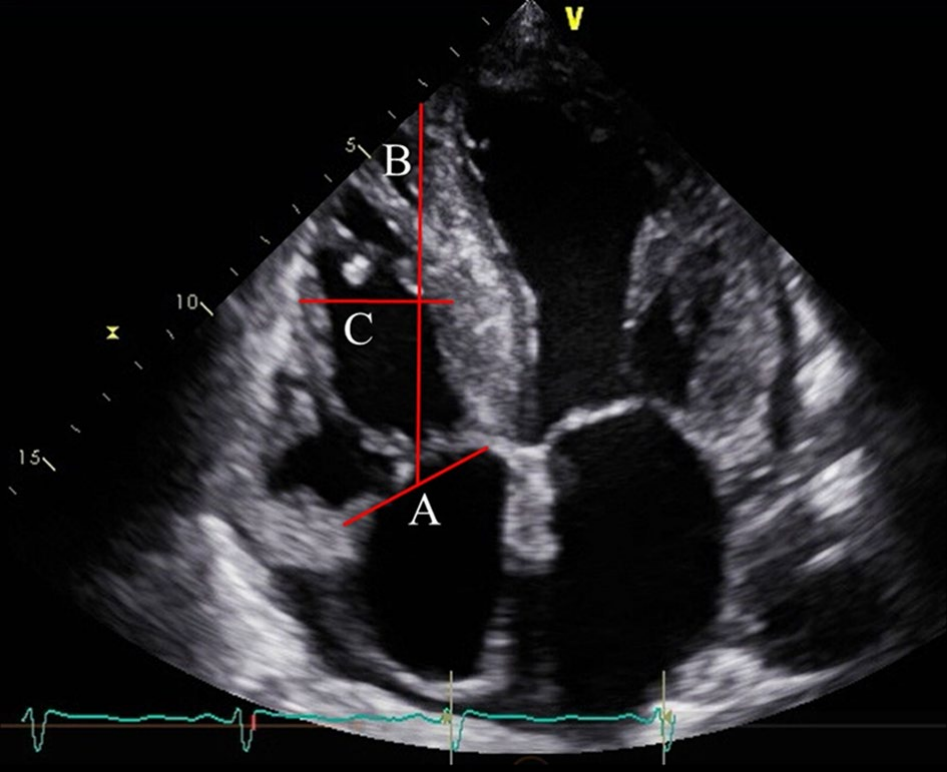
Definition of RV sphericity index. This echocardiographic image in apical four chamber view indicates measurement of RV sphericity index and tricuspid annular diameter. Initially, the tricuspid annulus diameter during the end-diastolic phase was measured (line A). Subsequently, a line was drawn connecting the RV apex to the midpoint of line A (line B). Finally, a perpendicular line was depicted connecting the RV free wall and septum, intersecting the midpoint of line B (line C). The RV sphericity index was calculated as the ratio between line C / line B. RV, right ventricular.

Echocardiography was conducted and analyzed by experienced cardiologists or clinical technologists. All patients underwent transthoracic echocardiography prior to TAVR. Severity of AS was determined based on the max velocity, mean pressure gradient, or AV area according to the ACC/AHA guideline^3^. LVEF, left ventricular end-diastolic volume (LVEDV), and left ventricular end-systolic volume (LVESV) were assessed by using modified Simpson’s biplane method. The right ventricular (RV) and atrial size and the diameter of the inferior vena cava were measured. RV sphericity index was calculated as the ratio between RV mid-ventricular and longitudinal diameters during the end-diastolic phase (Fig. 1)^12^, which is an indicator of RV remodeling^13^. LV sphericity index was also determined using same method as the RV sphericity index. Tricuspid annulus diameter was also measured during the end-diastolic phase. Systolic right ventricular function was assessed by TAPSE and RV fractional area change. Systolic pulmonary artery pressure (SPAP) was determined by measurement of the maximal tricuspid regurgitation velocity–derived gradient by continuous wave Doppler, inferior vena cava diameter, and respiratory-related changes in inferior vena cava diameter^14^. RV-PA coupling, which represents the association between the right ventricular contractility and pulmonary afterload, was defined as TAPSE-to-SPAP ratio (mm/mm Hg). Severity of mitral regurgitation (MR) and tricuspid regurgitation (TR) was scored on a scale ranging from 1 + (mild) to 4 + (severe)^15^ and 1 + (mild) to 5 + (torrential)^16^, respectively. None or trivial regurgitation was categorized as 0. MR and TR vena contracta (VC) were evaluated during the mid-systolic phase.

### Definitions

Reduced LVEF was defined as LVEF less than 40%. Pulmonary hypertension (PH) was determined if SPAP was 38 mmHg or higher according to transthoracic echocardiography^17^. Chronic kidney disease (CKD) stages 3B–5 was diagnosed based on an estimated glomerular filtration rate < 45 ml/min/1.73 m^2^^18^. Baseline laboratory evaluation of hepatobiliary function included total bilirubin (TB), alkaline phosphatase (ALP), and GGT. TB was considered abnormal if it exceeded 1.2 mg/dL, irrespective of sex. For ALP and GGT, we used sex-specific laboratory cutoff values as follows: ALP, 130 U/L (males) and 105 U/L (females); gamma glutamyl transferase (GGT), 59 U/L (males) and 39 U/L (females). Hepatobiliary system impairment was defined as elevation of at least two of three parameters (TB, ALP, and GGT)^19^.

Patients were classified based on aortic mean pressure gradient (dPmean), stroke volume index (SVi), and LVEF into four groups: (1) dPmean of ≥ 40 mmHg (high-gradient [HG]); (2) dPmean of < 40 mmHg and LVEF of < 50% (classical low-flow low-gradient [LFLG]); (3) dPmean of < 40 mmHg, LVEF of ≥ 50% and SVi of ≤ 35 ml/m^2^ (paradoxical LFLG)^20^; (4) dPmean of < 40 mmHg, LVEF of ≥ 50%, and SVi of > 35 mi/m^2^ (normal-flow low-gradient [NFLG]). Patients with classical LFLG underwent dobutamine stress echocardiography. The Clinical Frailty Scale was assessed as a marker of physical activity. This scale ranges 1 (very fit) to 9 (terminal ill), with a higher number indicating lower activity levels^21^. EuroSCORE II, which predicts in-hospital mortality after cardiac surgery, was calculated based on patient-related, cardiac-related, and operation-related factors^22^.

### Ethical statement

The study protocol conformed to the ethical guidelines of the 1975 Declaration of Helsinki. This study was approved by the Ethics Committee of our University on 11th July 2022 (the approval number: 22-087-B). The study satisfied the conditions needed to waive the requirement for written informed consent from the study participants. The ethics committee approved this waiver.

### Statistical analysis

Continuous variables are described as mean ± standard deviation if the skewness-kurtosis test did not reject the hypothesis of normality. Otherwise, variables are presented as medians with interquartile range values. Categorical variables are displayed as absolute numbers and percentages. Continuous variables were assessed using unpaired Student’s t tests or Mann–Whitney U tests, whereas Fisher’s exact test or the chi-squared test was used for categorical variables, as appropriate. Each cutoff value of TAPSE or RV sphericity index was determined based on the Liu index. The intra- and inter-observer reproducibility for the RV sphericity index were assessed through Bland–Altman analysis in thirty-four patients, respectively. Before the analysis, the skewness-kurtosis test was conducted to confirm whether the differences followed a normal distribution. When the RV sphericity index measured by X and Y, 100 (X–Y)/mean (X, Y) is plotted on the y-axis, mean (X, Y) on the x-axis. The risk of two-year MACCE was evaluated using Cox regression analysis and expressed as hazard ratio (HR) with 95% confidence interval (CI). The follow-up began on the day of TAVR. The follow-up was completed two years after the procedure or the date when the end-point events were observed, depending on what happened first. Multivariate Cox regression analysis was conducted using forward–backward stepwise selection. Variables with a *p* value < 0.25 in the univariate Cox regression analysis were selected for the multivariate Cox regression analysis. A minimum of five outcome events per predictor variable was applied to the construction of multivariate models^23^. The nonparametric bootstrap method, resampling with replacement 1000 times, was conducted to provide inner validation. Statistical significance was defined as a *p* value < 0.05. All statistical analyses were performed using Stata version 14 (StataCorp, College Station, TX, USA).

## Results

### Patient characteristics

After exclusion of 5 patients following TAVR in SAV for AR, this study included a total of 239 patients (age, 86 ± 5 years; males, 32%). HG was present in 161 (67%), classical LFLG in 29 (12%), paradoxical LFLG in 39 (16%), and NFLG in 10 (4%). Patient characteristics are shown in Table 1. Previous myocardial infarction and concomitant congestive heart failure were observed in 19 (8%) and 122 (51%). CKD stages 3B–5, hepatobiliary system impairment, peripheral vascular disease, cerebrovascular disease, and chronic obstructive pulmonary disease were identified in 91 (38%), 20 (8%), 60 (25%), 29 (12%), and 34 (14%), respectively. EuroSCORE II and clinical frailty score were 3.38 (2.12–4.63) and 4 (3–5). LVEF, LVEDV, and LVESV were 58 ± 11%, 72 (59–94) ml, and 28 (22–43) ml. LVEF of < 40% was observed in 20 patients (8%).

**Table 1:** Patient characteristics.

	All (n = 239)
Age, years	86 ± 5
Male, n (%)	77 (32)
Body mass index, kg/m^2^	22 ± 4
Diabetes mellitus, n (%)	63 (26)
Dyslipidemia, n (%)	128 (54)
Hypertension, n (%)	168 (70)
Chronic kidney disease stage 3B or higher, n (%)	91 (38)
Hepatobiliary system impairment, n (%)	20 (8)
Previous myocardial infarction, n (%)	19 (8)
Coronary artery disease, n (%)	95 (40)
Congestive heart failure, n (%)	122 (51)
Atrial fibrillation, n (%)	54 (23)
Peripheral vascular disease, n (%)	60 (25)
Cerebrovascular disease, n (%)	29 (12)
Chronic obstructive pulmonary disease, n (%)	34 (14)
Hemodynamic classification	
High-gradient, n (%)	161 (67)
Classical Low-flow low-gradient, n (%)	29 (12)
Paradoxical Low-flow low-gradient, n (%)	39 (16)
Normal-flow low-gradient, n (%)	10 (4)
EuroSCORE II	3.38 (2.12–4.63)
Clinical frailty score	4 (3–5)
Medication prior to TAVR	
RAS inhibitors, n (%)	119 (50)
ARNI, n (%)	5 (2)
Beta blockers, n (%)	109 (46)
MRA, n (%)	69 (29)
Loop diuretics, n (%)	117 (49)
Statin, n (%)	126 (53)
SGLT2 inhibitors, n (%)	10 (4)
Medication at discharge	
RAS inhibitors, n (%)	106 (44)
ARNI, n (%)	7 (3)
Beta blockers, n (%)	79 (33)
MRA, n (%)	155 (65)
Loop diuretics, n (%)	185 (77)
Statin, n (%)	125 (52)
SGLT2 inhibitors, n (%)	9 (4)
Single antiplatelet therapy, n (%)	144 (60)
Dual antiplatelet therapy, n (%)	53 (22)
Direct oral anticoagulation, n (%)	52 (22)
Vitamin K antagonists, n (%)	11 (5)
Neither antiplatelet therapy nor anticoagulation, n (%)	13 (5)
*Laboratory data*	
Hemoglobin, g/dL	11.0 ± 1.7
Albumin, g/dL	3.7 ± 0.5
Creatinine, mg/dL	0.9 (0.7–1.1)
eGFR, ml/min/1.73m2	50 (39–64)
B-type natriuretic peptide, pg/mL	172 (83–366)
C-reactive protein, mg/dl	0.1 (0.1–0.5)
*Echocardiography*	
AV maximum PG, mmHg	78 ± 24
AV mean PG, mmHg	47 ± 16
AV area, cm^2^	0.7 ± 0.2
LVEDV, ml	72 (59–94)
LVESV, ml	28 (22–43)
LV sphericity index	0.50 (0.45–0.54)
LVEF, %	58 ± 11
E/e’	14.0 (11.1–19.9)
MR severity, n (%)	
0	44 (18)
1+	167 (70)
2+	25 (10)
3+	2 (1)
4+	1 (0)
MR vena contracta, mm	1.8 (0.9–2.8)
RV longitudinal diameter, mm	65 ± 9
RV mid-ventricular diameter, mm	23 ± 4
RV sphericity index	0.36 (0.31–0.39)
Tricuspid annulus diameter, mm	32 ± 5
RA area, cm^2^	15 ± 9
TAPSE, mm	20 ± 3
RV fractional area change, %	45 ± 10
SPAP, mmHg	33 ± 10
RV-PA coupling, mm/mmHg	0.71 ± 0.31
TR severity, n (%)	
0	32 (13)
1+	155 (65)
2+	39 (16)
3+	12 (5)
4+	1 (0)
5+	0 (0)
TR vena contracta, mm	2.0 (1.4–3.0)
*Procedure*	
TAVR access route	
Transfemoral access, n (%)	221 (92)
Transapical access, n (%)	13 (5)
Transsubclavian/transaxillary access, n (%)	5 (2)

ARNI, angiotensin receptor neprilysin inhibitor; AV, aortic valve; eGFR, estimated glomerular filtration rate; LVEDV, left ventricular end-diastolic volume; LVEF, left ventricular ejection fraction; LVESV, left ventricular end-systolic volume; MR, mitral regurgitation; MRA, mineralocorticoid receptor antagonists; PA, pulmonary artery; PG, pressure gradient; RA, right atrium; RAS, renin–angiotensin system; RV, right ventricle; SGLT2, sodium-glucose cotransporter-2; SPAP, systolic pulmonary artery pressure; TAPSE, tricuspid annular plane systolic excursion; TR, tricuspid regurgitation.

TAPSE and SPAP were 20 ± 3 mm and 33 ± 10 mmHg. SPAP was not evaluated in ten patients because of no TR. RV-PA coupling was 0.71 ± 0.31. RV longitudinal diameter, RV mid-ventricular diameter, RV sphericity index, and tricuspid annulus were 65 ± 9 mm, 23 ± 4 mm, 0.36 (0.31–0.39), and 32 ± 5 mm, respectively. PH based on echocardiography was identified in 58 individuals (25%). MR and TR severity of ≥ 3 + were observed in 3 (1%) and 13 (5%).

### Clinical impact of the left and right heart structure and function

**Figure 2:**
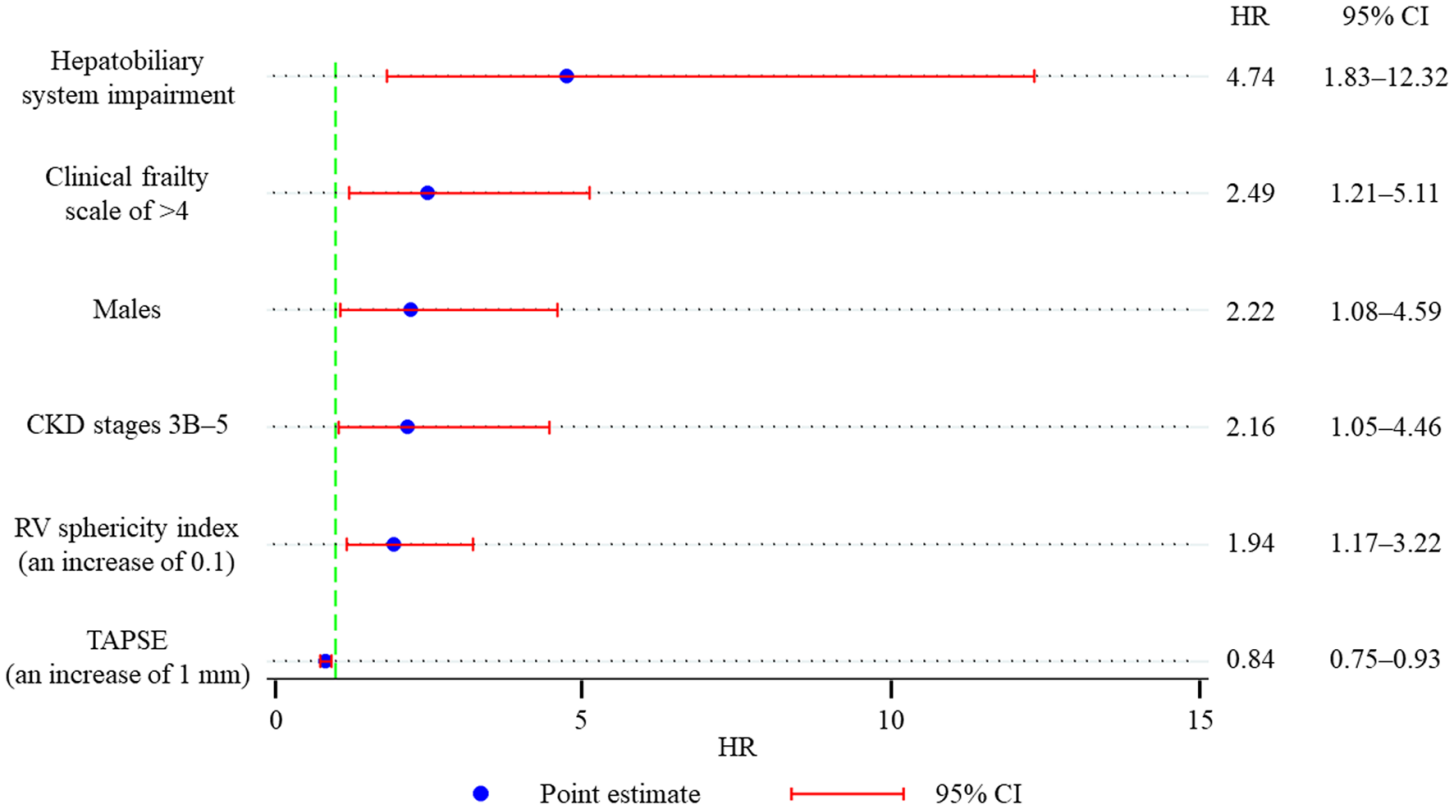
Forest plot of the multivariate Cox regression analysis for two-year MACCE. The multivariate Cox regression analysis demonstrated that lower TAPSE, higher RV sphericity index, hepatobiliary system impairment, CKD stages 3B–5, males, and clinical frailty scale of > 4 were associated with higher 2-year MACCE. CI, confidence interval; CKD, chronic kidney disease; HR, hazard ratio; MACCE, major adverse cardiac and cerebrovascular events; RV, right ventricular; TAPSE, tricuspid annular plane systolic excursion.

**Figure 3:**
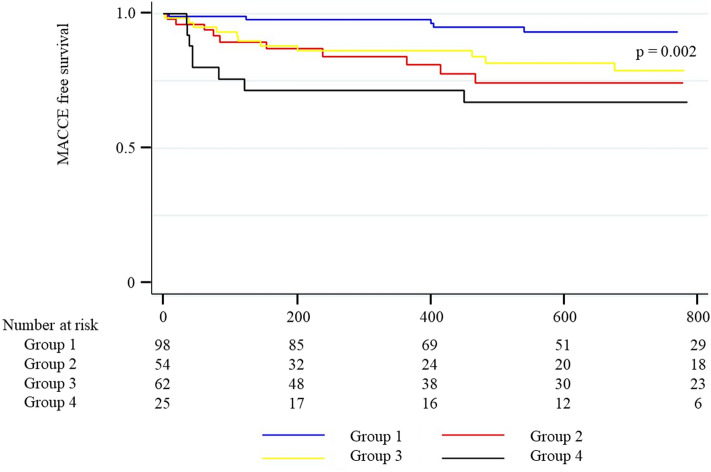
Clinical impact of TAPSE and RV sphericity index on 2-year MACCE. The patients were classified into group 1 (TAPSE of ≥ 19 mm and RV sphericity index of < 0.377), group 2 (TAPSE of < 19 mm and RV sphericity of < 0.377), group 3 (TAPSE of ≥ 19 mm and RV sphericity index of ≥ 0.377), and group 4 (TAPSE of < 19 mm and RV sphericity index of < 0.377). Kaplan–Meier survival curve demonstrated that the incidence of MACCE at 2 years differed among the above groups (*p* = 0.002). Patients with abnormal values of TAPSE and RV sphericity index (group 4) indicated the highest incidence of the outcomes. MACCE, major adverse cardiac and cerebrovascular events; RV, right ventricular; TAPSE, tricuspid annular plane systolic excursion.

Two-year MACCE was observed in 34 (14%) and follow-up duration was 556 (220–925) days. Of eligible patients, lost to follow up was observed in 33 patients (14%). Table 2 discloses the results of univariate Cox regression analysis for two-year MACCE. According to the univariate analysis, both LV and RV parameters predicted the outcomes. However, the multivariate Cox regression analysis excluded the LV parameter and demonstrated that echocardiographic predictors were TAPSE (adjusted HR in an increase of 1 mm, 0.84; 95% CI, 0.75–0.93; *p* = 0.001) and RV sphericity index (adjusted HR in an increase of 0.1, 1.94, 95% CI, 1.17–3.22; *p* = 0.010) (Fig. 2). Bootstrap method provided the similar results (TAPSE: adjusted HR in an increase of 1 mm, 0.84; 95% CI, 0.75–0.94; *p* = 0.002; RV sphericity index: adjusted HR in an increase of 0.1, 1.94; 95% CI, 1.12–3.36; *p* = 0.018). Cutoff values ant the *C*-statistics of TAPSE and RV sphericity index were 19 mm (0.65 [0.56–0.74]) and 0.377 (0.60 [0.49–0.71]), respectively. Patients with at least an impaired RV parameters underwent a higher incidence of two-year MACCE compared to those without such disorders (Fig. 3). RA area, SPAP, and TR severity were not correlated with the clinical adverse events. It is noteworthy that RV-PA coupling did not predict the worse outcomes.

**Table 2:** Univariate Cox regression analysis for two-year MACCE.

	Univariate analysis		
	HR	95% CI	p value
Age, an increase of a year	0.98	0.91–1.05	0.500
Male	1.68	0.84–3.33	0.140
Body mass index, an increase of 1 kg/m^2^	0.90	0.82–0.99	0.029
Diabetes mellitus	0.86	0.39–1.90	0.714
Dyslipidemia	0.91	0.47–1.79	0.791
Hypertension	1.37	0.62–3.03	0.434
Chronic kidney disease stage 3B or higher	2.04	1.04–4.01	0.038
Hepatobiliary system impairment	3.29	1.35–8.00	0.009
Previous myocardial infarction	0.78	0.19–3.26	0.734
Coronary artery disease	0.84	0.42–1.70	0.625
Congestive heart failure	2.59	1.24–5.42	0.012
Atrial fibrillation	2.04	1.00–4.15	0.049
Peripheral vascular disease	0.98	0.44–2.17	0.963
Cerebrovascular disease	1.64	0.68–3.97	0.270
Chronic obstructive pulmonary disease	0.39	0.09–1.64	0.199
Hemodynamic classification			
High-gradient		Reference	
Classical low-flow low-gradient	3.91	1.75–8.73	0.001
Paradoxical low-flow low-gradient	0.95	0.32–2.80	0.924
Normal-flow low-gradient	2.96	0.87–10.04	0.082
EuroSCORE II, an increase of 1 point	1.09	1.01–1.18	0.020
Clinical frailty score > 4	2.19	1.10–4.33	0.025
Medication at discharge			
RAS-I	0.94	0.48–1.86	0.863
ARNI	1.49	0.20–10.92	0.696
Beta blockers	2.96	1.50–5.83	0.002
MRA	1.15	0.55–2.41	0.709
Loop diuretics	1.16	0.48–2.80	0.742
Statin	1.09	0.56–2.14	0.800
SGLT2 inhibitors	0.72	0.10–5.30	0.750
Antiplatelet therapy			
No antiplatelet therapy		Reference	
Single antiplatelet therapy	0.61	0.28–1.32	0.208
Dual antiplatelet therapy	0.34	0.11–1.10	0.071
Anticoagulation	1.59	0.79–3.22	0.194
Laboratory data			
Hemoglobin, an increase of 1.0 g/dL	1.05	0.86–1.27	0.636
Albumin, an increase of 1.0 g/dL	0.52	0.26–1.05	0.068
Creatinine, an increase of 1.0 mg/dL	1.78	1.20–2.64	0.004
eGFR, an increase of 10 ml/min/1.73m2	0.84	0.69–1.02	0.078
B-type natriuretic peptide, an increase of 1000 pg/mL	1.72	0.73–4.05	0.215
C-reactive protein, an increase of 1.0 mg/dl	1.06	0.90–1.24	0.505
*Echocardiography*			
LVEDV, an increase of 10 ml	1.03	0.92–1.15	0.617
LVESV, an increase of 10 ml	1.12	0.96–1.31	0.161
LV sphericity index, an absolute increase of 1%	1.84	0.01–283.01	0.813
LVEF, an absolute increase of 10%	0.73	0.56–0.95	0.021
E/e’, an absolute increase of 1%	1.02	0.98–1.07	0.331
RV longitudinal diameter, an increase of 10 mm	0.69	0.52–0.93	0.016
RV mid-ventricular diameter, an increase of 1 mm	1.02	0.94–1.11	0.563
RV sphericity index, an increase of 0.1	1.45	0.89–2.39	0.139
RV sphericity index of > 0.377	2.18	1.11–4.30	0.024
Tricuspid annulus diameter, an increase of 1 mm	1.04	0.98–1.11	0.201
RA area, an increase of 1 cm^2^	1.02	0.99–1.05	0.138
TAPSE, an increase of 1 mm	0.85	0.76–0.94	0.002
TAPSE of < 19 mm	2.72	1.38–5.33	0.004
RV fractional area change, an absolute increase of 1%	0.97	0.93–1.00	0.085
SPAP, an increase of 1 mmHg	1.01	0.97–1.04	0.732
RV-PA coupling, an increase of 1 mm/mmHg	0.44	0.10–1.92	0.273
MR ≥ 3 +	2.97	0.41–21.73	0.284
TR ≥ 3 +	1.84	0.56–6.03	0.312
Transapical access (reference: transfemoral access)	1.74	0.53–5.70	0.360

ARNI, angiotensin receptor neprilysin inhibitor; AV, aortic valve; CI, confidence interval; eGFR, estimated glomerular filtration rate; HR, hazard ratio; LVEDV, left ventricular end-diastolic volume; LVEF, left ventricular ejection fraction; LVESV, left ventricular end-systolic volume; MACCE, major adverse cardiac and cerebrovascular events; MR, mitral regurgitation; MRA, mineralocorticoid receptor antagonists; PA, pulmonary artery; PG, pressure gradient; RA, right atrium; RAS, renin–angiotensin system; RV, right ventricle; SGLT2, sodium-glucose cotransporter-2; SPAP, systolic pulmonary artery pressure; TAPSE, tricuspid annular plane systolic excursion; TR, tricuspid regurgitation.

### Predictors other than echocardiographic parameters for 2-year MACCE

As shown in Table 2, BMI, CKD stages 3B–5, hepatobiliary system impairment, concomitant CHF, classical LFLG, and clinical frailty score of > 4 predicted the clinical adverse events. The multivariate Cox regression analysis identified noncardiac parameters such as CKD stages 3B–5 (adjusted HR, 2.16; 95% CI, 1.05–4.46; *p* = 0.036), hepatobiliary system impairment (adjusted HR, 4.74; 95% CI, 1.83–12.32; *p* = 0.001), male sex (adjusted HR, 2.22; 95% CI, 1.08–4.59; *p* = 0.031), and clinical frailty score of > 4 (adjusted HR, 2.49; 95% CI, 1.21–5.11; *p* = 0.013) (Fig. 2).

### Association between the left and right heart structure and function

**Figure 4:**
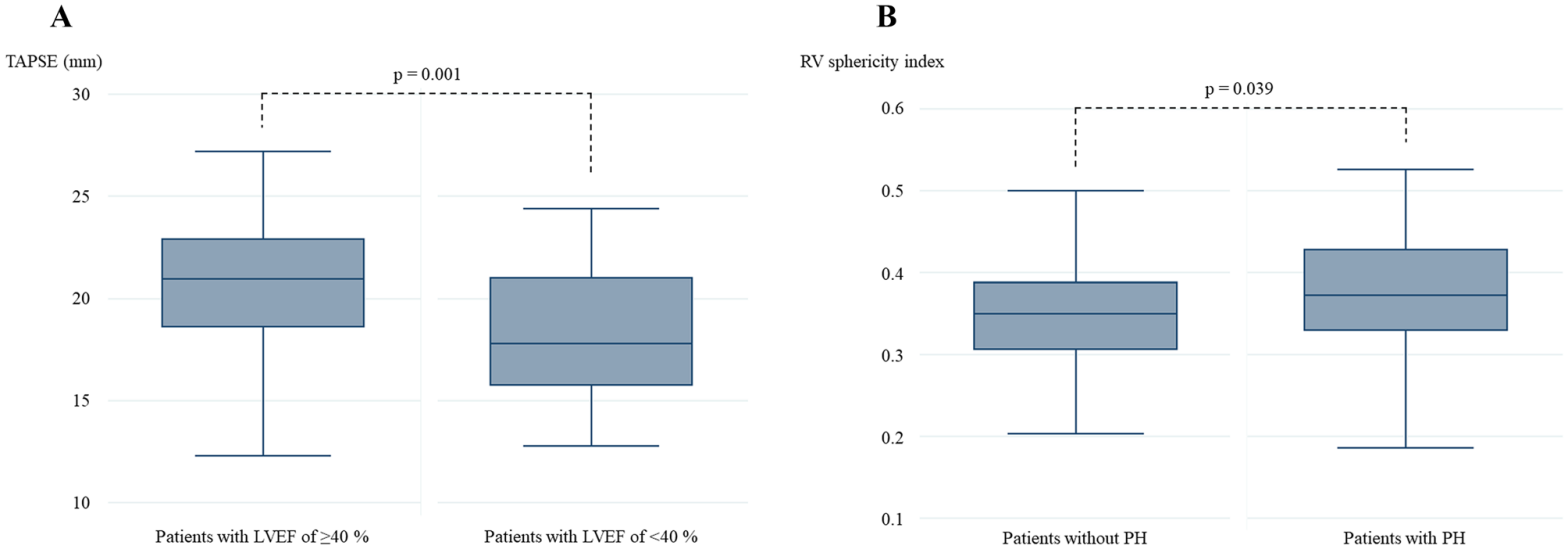
A comparison of the LV and RV characteristics. (**A**) A value of TAPSE was higher in patients with LVEF of ≥ 40%. (**B**) A value of RV sphericity index was higher in patients with PH. LV, left ventricular; LVEF, left ventricular ejection fraction; PH, pulmonary hypertension; RV, right ventricular; TAPSE, tricuspid annular plane systolic excursion.

There was a significant difference of TAPSE between patients with and without reduced LVEF (*p* = 0.001) (Fig. 4A). PH was not associated with a value of TAPSE (*p* = 0.624). On the other hand, PH was significantly related to higher RV sphericity index (*p* = 0.039) (Fig. 4B) and reduced LVEF was not (*p* = 0.224). Larger tricuspid annulus diameters and RA areas were observed in patients with PH (*p* = 0.002 and *p* < 0.001, respectively). Association of reduced LVEF with such parameters was not observed (*p* = 0.642 and *p* = 0.211, respectively).

### Reproducibility of the RV sphericity index

**Figure 5:**
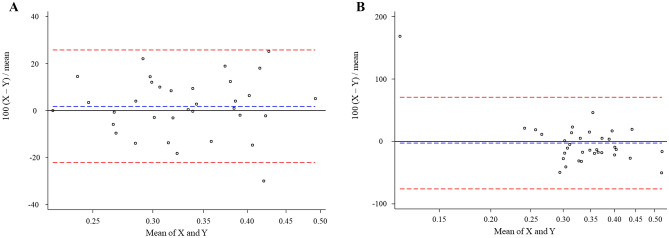
Bland Altman analysis for reproducibility of the RV sphericity index. The blue dashed line indicates the mean and the red dashed lines represent the 95% limits of agreement. (**A**) The Bland Altman plot depicts good intra-observer agreement. X: The second measurement by S.H. Y: The first measurement by S.H. (**B**) This figure demonstrates good inter-observer agreement. X: measurement by S.H. Y: measurement by H.M. RV, right ventricular.

The intra- and inter-observer differences were − 0.01 ± 0.04 and − 0.02 ± 0.07, both of which followed a normal distribution according to the skewness-kurtosis test (*p* = 0.115 and *p* = 0.468, respectively). Figure 5 demonstrates good intra- and inter-observer agreement.

## Discussion

The current study demonstrated that RV structural and functional parameters such as RV sphericity index and TAPSE predicted the subsequent MACCE, whereas those of LV did not. Further, a lower value of TAPSE was associated with reduced LVEF and a higher value of RV sphericity index was correlated with PH. Noncardiac comorbidities such as impaired kidney function and hepatobiliary system impairment were also clinical adverse predictors. Although severe AS is the most common left-sided valve lesion, its prognosis can be determined by other lesions. Considering that the RV dysfunction could be a result of advanced LV dysfunction^10^, early intervention may be needed before progression of advanced heart failure.

Although the RV has been recently regarded as an important predictor in cardiovascular diseases, there are few investigations evaluating RV structural characteristics, probably due to the complexity. The LV has an ellipsoid-shaped chamber surrounded by relatively thick musculature, whereas the RV has a crescent-shaped chamber with a thin wall^24^. Further, multiple interactions between the LV and RV make the interpretation of the RV structural findings much more challenging^25^. The unique features make accurate assessment of RV structure difficult; however, some parts of the RV such as shape and tricuspid annulus diameter may reflect its structural disorder to some extent. A previous single-center study refereed to CT-determined tricuspid annulus as a useful predictor^5^. Our study evaluated RV sphericity index as well as tricuspid annulus diameter as a marker of an abnormal RV structure. Consequently, only the former predicted the adverse clinical events. This result would make sense because RV sphericity index evaluates both RV longitudinal and transverse diameters, whereas tricuspid annulus diameter reflects a transverse diameter. RV sphericity index might reflect RV remodeling considering that PH was associated with higher RV sphericity index. Association between TAPSE and LV systolic function has been reported previously^26^. RV dysfunction may be a direct result of LV impairment^27^, which could be mediated by the largely septum, but also by LV free wall^28^. On the other hand, RV dysfunction can also impair LV function by attenuating LV preload and adversely affecting the systolic and diastolic interaction via the intraventricular septum and the pericardium^26^. The potential bidirectional influences mentioned above can help explain why the LV has a lesser impact on two-year MACCE compared to the RV. The clinical impact of the LV features was attenuated in patients with advanced heart failure^14^.

The results of our study recommend assessment of RV characteristics for a risk stratification. The current study evaluated RV features based on 2-dimensional (2D) images, which may be less accurate than 3-dimensional (3D) images. Indeed, a recent study indicated a predictive ability of 3D RV ejection fraction was superior to 2D evaluation such as TAPSE and fractional area change^29^. However, a 3D-image construction of the RV is not available in some cases because of technical difficulty. Cardiovascular magnetic resonance (CMR) can provide 3D RV images easily and more accurate information regarding structure and function compared to echocardiography^30^. It is difficult to apply CMR to all patients scheduled for TAVR in daily clinical practice. Therefore, the RV structure and function should be evaluated by echocardiography at first to identify patients with obviously normal RV structure and function. It may make sense that CMR is applied for patients in whom echocardiography suggests the RV impairment or sufficient evaluation is difficult.

No association of RV-PA coupling with MACCE could be owing to a higher prevalence of heart failure patients. SPAP might become lower after removal of afterload mismatch and pulmonary congestion. According to a previous study, SPAP decreased after the procedure, whereas RV systolic function did not significantly improve^6^. Another previous study indicated that RV-PA coupling prior to TAVR did not predict long-term mortality^31^.

Noncardiac predictors such as hepatobiliary system impairment and renal impairment may be modifiable comorbidities in selective patients. However, considering that SPAP was not associated with the clinical adverse events, modifiable parameters did not predict the outcomes necessarily. It is occasionally difficult to distinguish “true” CKD from cardiorenal syndrome. The same can be said for hepatobiliary system impairment. Future studies dedicated to investigation of such comorbidities would be needed for expanding understanding of prognosis in patients with severe AS.

### Limitations

There are several limitations to be addressed. At first, this was a single-center study. Because of its nature, a selection bias might affect our results. Second, the sample size might not large enough. However, the similar result derived from the bootstrap method reinforced the reliability of our findings. Third, our study did not include strain analysis, which may contribute to better risk stratification compared to TAPSE or fractional area change. Finally, as mentioned above, the RV structure and function were not evaluated by CMR. We believe that echocardiographic findings in the present study are reliable; however, CMR would demonstrate much more accurate findings.

## Conclusions

The RV sphericity index and TAPSE predicted two-year MACCE, whereas the LV parameters did not. The incidence of the adverse clinical events was dependent on the RV and noncardiac characteristics. Severe AS is the left-sided heart disease; however, its prognosis is determined by the other lesions.

## Data Availability

The data supporting the findings of this study will be shared on reasonable request to the corresponding author.
